# Supplementary material for: Diverse MR1-restricted T cells in mice and humans
Source: Nat Commun. 2019 May 21;10:2243. doi: 10.1038/s41467-019-10198-w (PMC6529461; doi:10.1038/s41467-019-10198-w)
Supplement: Supplementary file 1 — Supplementary Information [file 41467_2019_10198_MOESM1_ESM.pdf]

**Supplementary Information PDF**

**Koay et al., Diverse MR1-restricted T cells in mice and humans.**

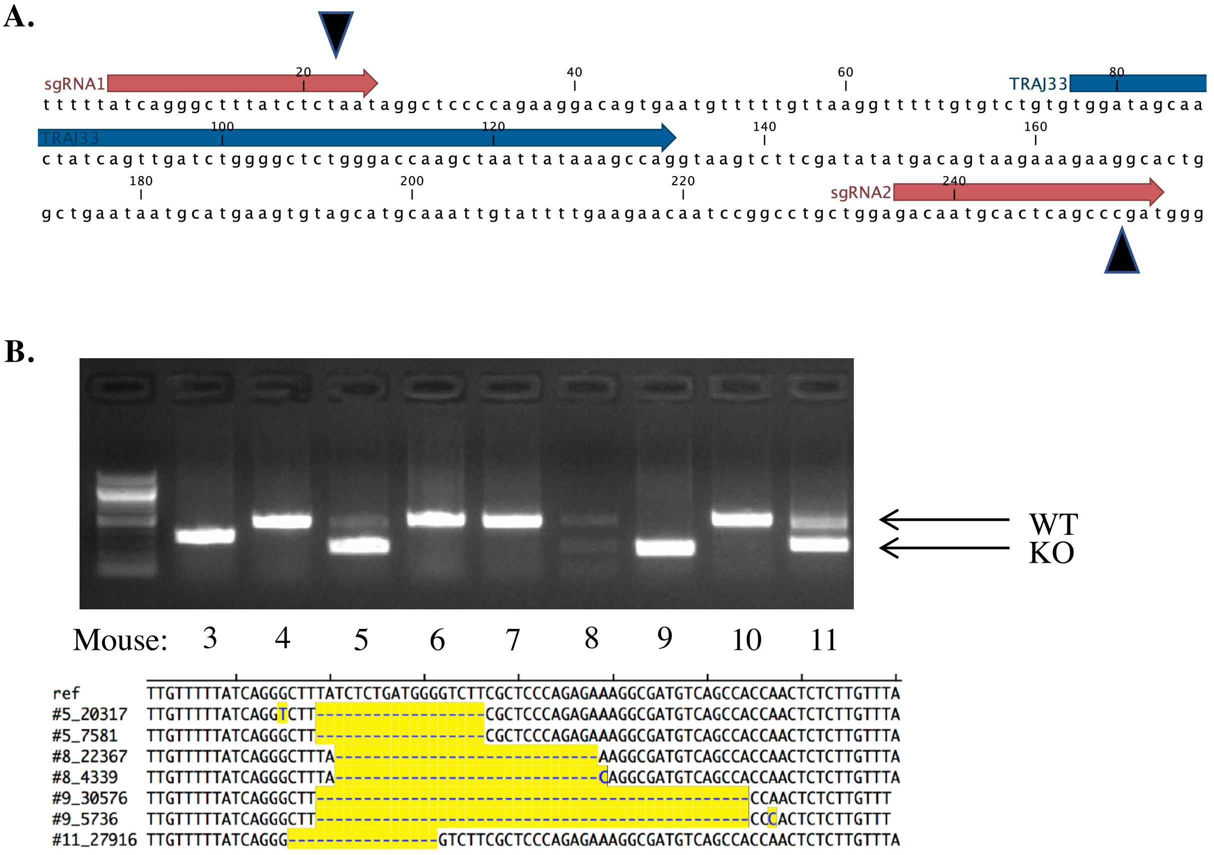


**Supplementary Figure 1. Generation of *Traj33* deficient mice.**

**A.** *Traj33* KO mice were generated by CRISPR/Cas9-mediated deletion of the *Traj33* gene in C57BL/6 blastocysts using flanking sgRNA motifs, as per [46]. **B.** PCR of *Traj33* on chimeric founder mice, showing the WT and *Traj33*KO amplicons. Genomic InDel sequencing analysis of founder mice.

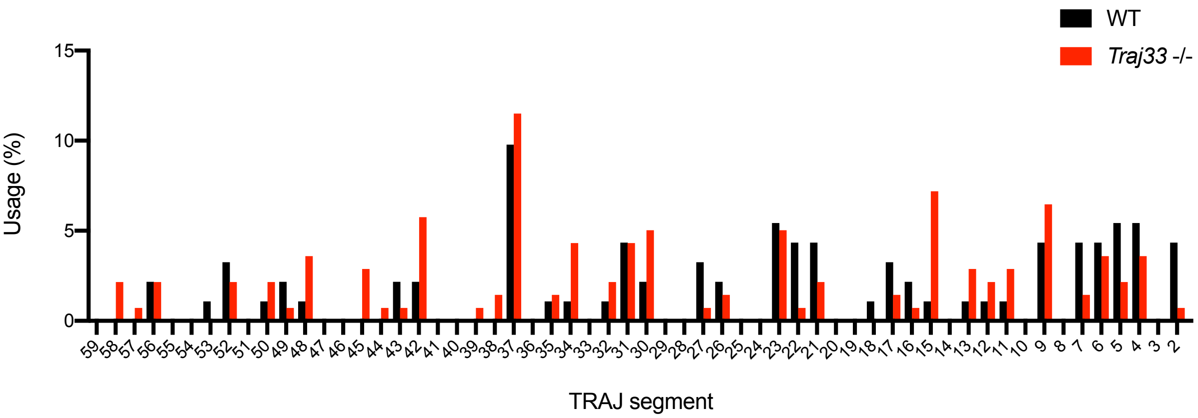


| | Downstream of *Traj33* | Upstream of *Traj33* |
|---|---|---|
| WT | 63 | 29 |
| *Traj33* KO | 79 | 60 |
| p= 0.097 | | |

**Supplementary Figure 2. Similar TCR Jα distribution in WT and *Traj33* deficient mice.**
**A.** PCR analysis for Vα (TRAV) and Jα (TRAJ) gene segments in 92 and 139 single-cell sorted double-positive thymocytes from WT and *Traj33*$^{-/-}$ mice respectively. Graph illustrates the frequency of use of genes encoding Ja rearrangements regardless of TRAV usage.

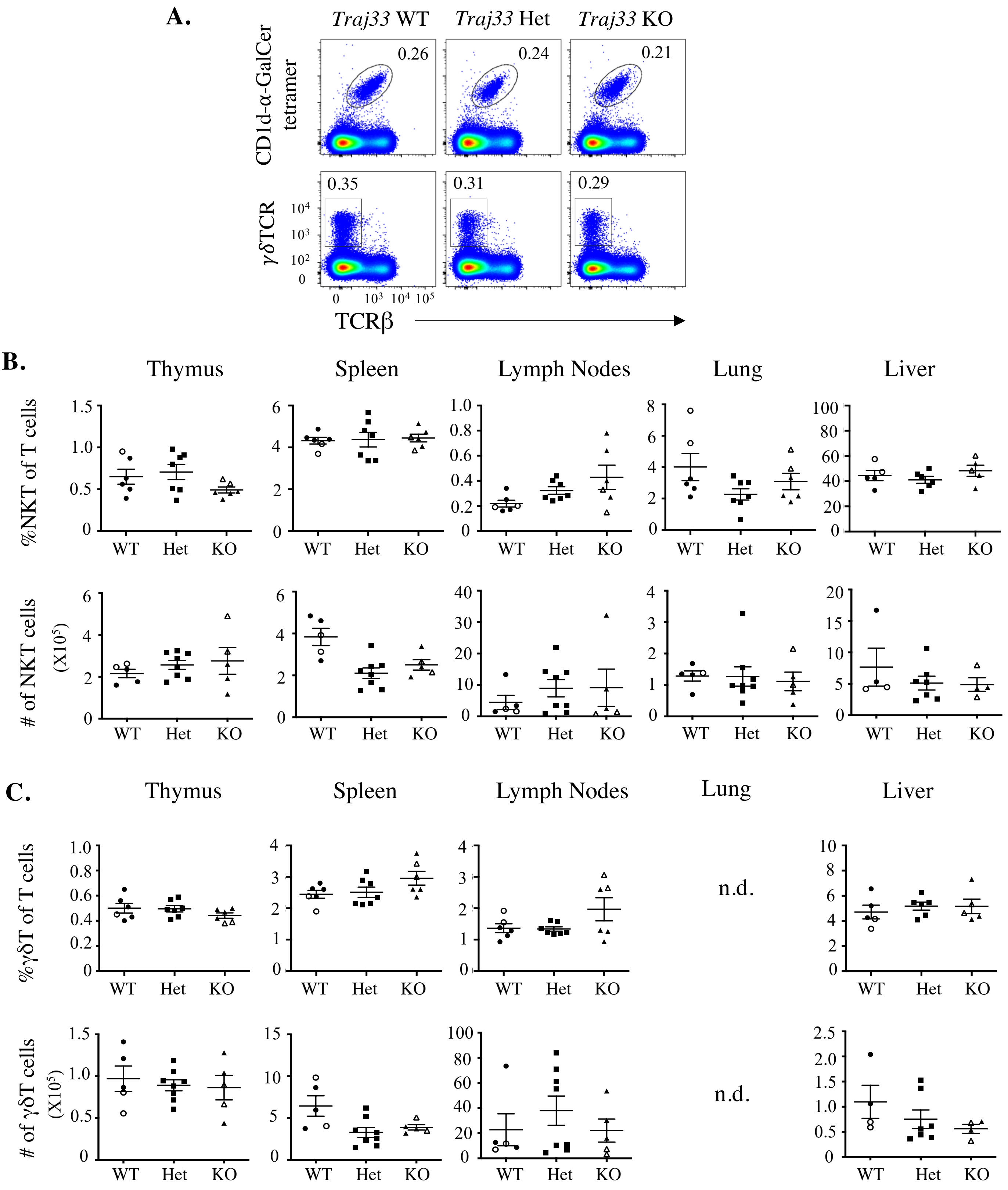


**Supplementary Figure 3. NKT cells and γδ T cells are unaffected in *Traj33* deficient mice**.

**A.** Flow cytometry analysis of CD1d-α-GalCer tetramer+ TCRβ$^+$ NKT and γδ T cells from thymus, from *Traj33* wild-type (WT), *Traj33* heterozygous (het) and *Traj33* knockout (KO) mice. Plots were pre-gated on B220- live lymphocytes, hence numbers show percentage of gated cells of B220- cells.

**B and C.** Frequencies of all T cells and absolute numbers of NKT cells **(B)** and γδ T **(C)** cells from thymus, anti-CD24-complement-depleted enriched thymus, spleen, lymph node, lung and liver from *Traj33* wild-type (WT), *Traj33* heterozygous (het) and *Traj33* knockout (KO) mice. Horizontal bars on scatter points signify mean ± SEM. Each scatter point represents an individual mouse, data from 3 independent experiments with a total of 4-8 mice per strain. Open symbols represent ex-breeders, >20 weeks old.

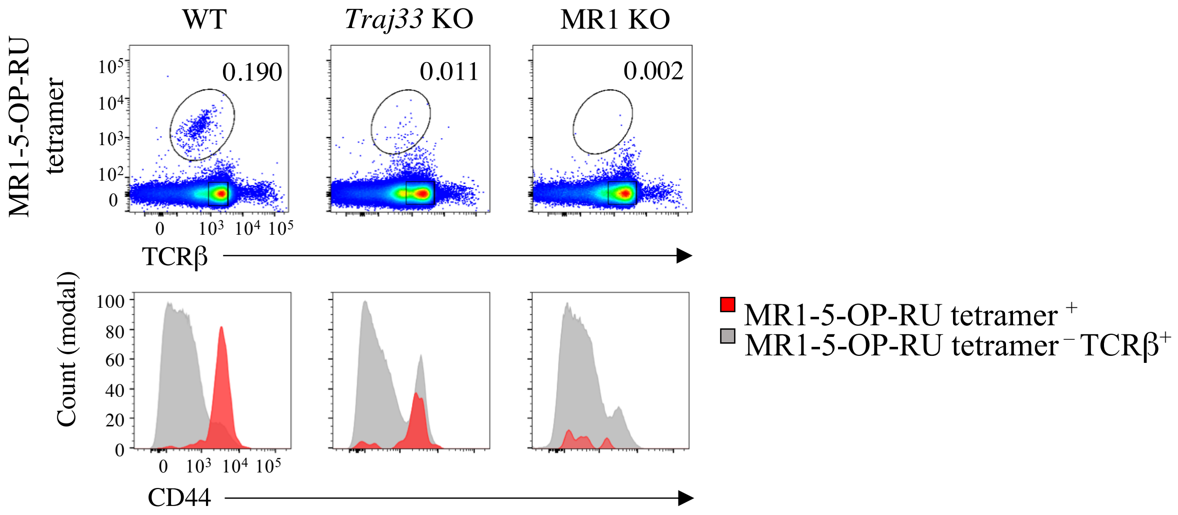


**Supplementary Figure 4.** Absence of mature CD44⁺ MR1-5-OP-RU tetramer⁺ cells in MR1-deficient mouse thymi (anti-CD24 depleted thymocytes).

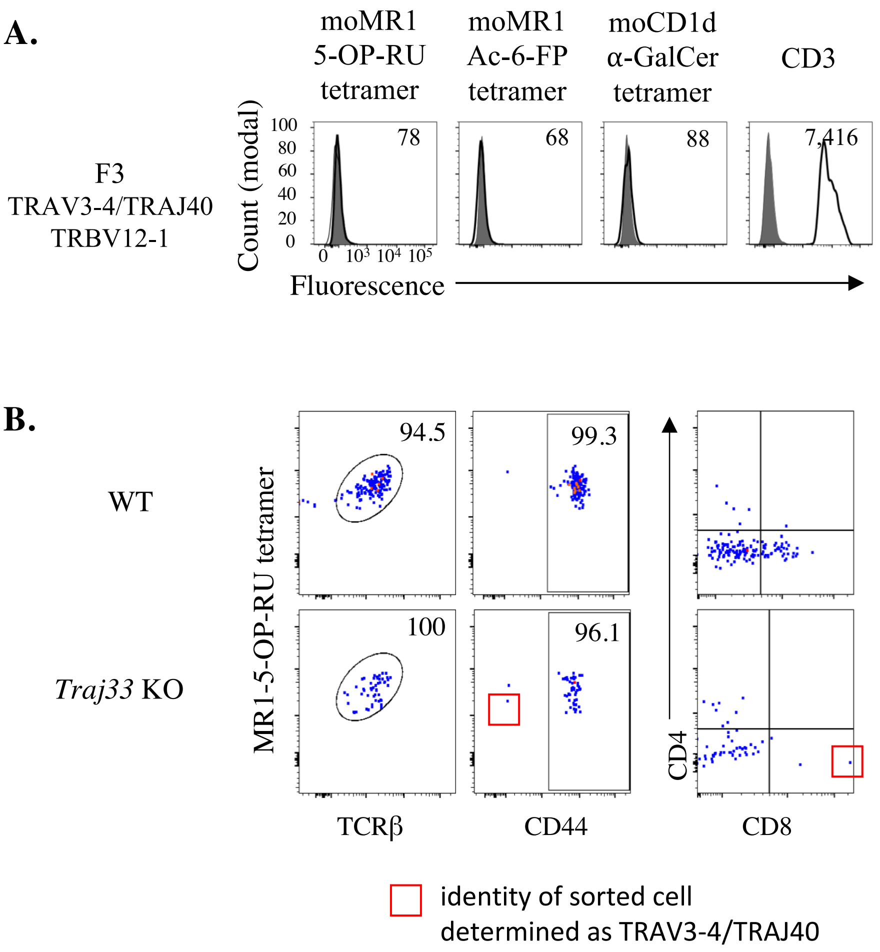


**Supplementary Figure 5. TRAV3-4/TRAJ40 failed to bind any MR1-Ag-tetramers.**
**A.** Paired TCR (TRAV3-4/TRAJ40 with TRBV12-1) was produced as a transiently transfected surface TCR on HEK293T cells. Flow cytometric histogram overlays depict staining intensity of these transfected HEK293T cells, stained with MR1-5-OP-RU, MR1-Ac-6-FP and CD1d-α-GalCer tetramers. Cells were pre-gated for high levels of GFP and surface TCR, and staining MFI of gated population (transparent histograms) is depicted in top right of each histogram, overlaid on unstained cells (grey histograms). **B**. Index-sort gating of MR1-5-OP-RU tetramer+ cells from WT and *Traj33* KO anti-CD24-complement-depleted enriched thymus (Figure 3) with red boxes indicating expression of MR1-tetramer, TCRβ, CD44, and CD8 for index sorted cell identified as TRAV3-4 TRAJ30.

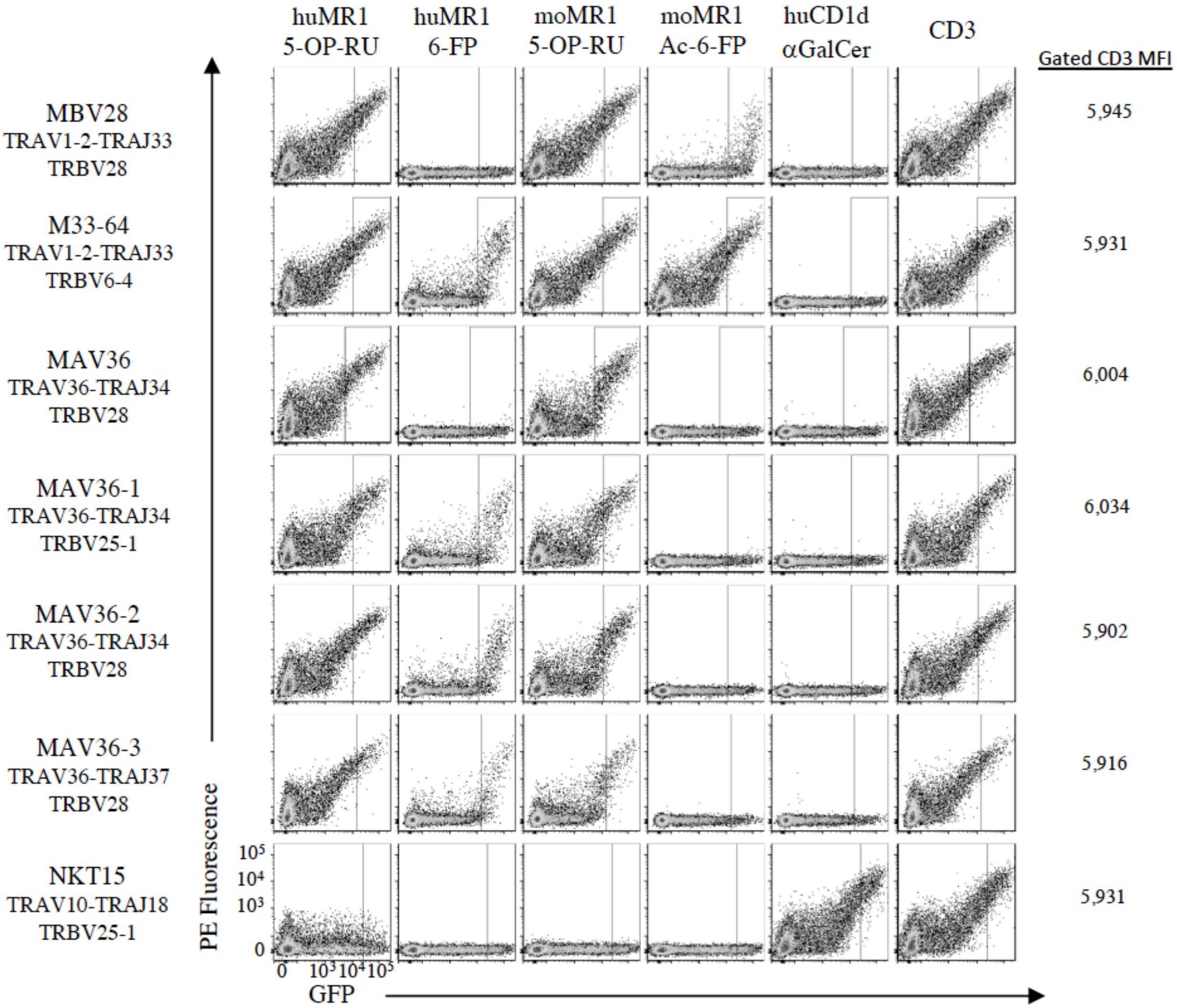


**Supplementary Figure 6. Gating of HEK293T transient transfection.** Flow cytometry plots showing MR1 (columns 1-5), CD1d (column 6) tetramer and CD3 (column 7) staining versus GFP on HEK293T cells transiently transfected to express human TCRs (left column). Cells are gated for viable 293T cells, MFI of gated CD3+ population is depicted on right of plots. Gates used to derive data in main manuscript figure 5 are shown on each graph.

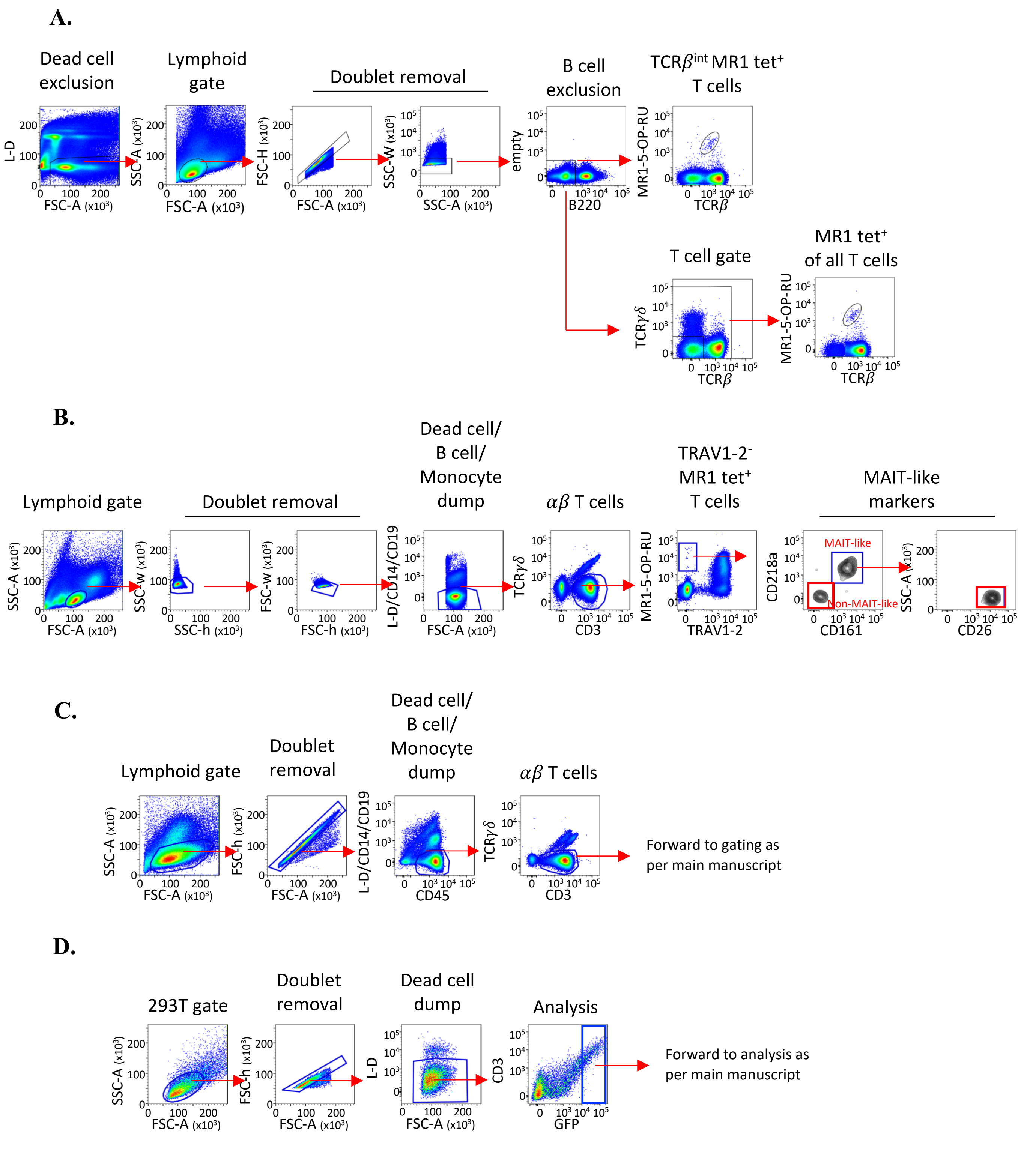


**Supplementary Figure 7. Flow cytometry gating strategies.** Flow cytometry plots showing sequential gating strategy. **(A)** depicts gating for detecting MR1-5-OP-RU$^{+}$ TCRβ$^{+}$ cells in mouse organs and for determination of MR1-tetramer$^{+}$ cell frequency of all mouse T cells, **(B)** depicts gating for scSorting of human PBMCs with sort gates shown in red boxes, **(C)** depicts gating for analysis of surface markers and transcription factors on human PBMCs, and **(D)** depicts gating of HEK293T cells for transient transfections.

**Supplementary Table 1.** TCR sequences of *in vitro* antigen-expanded MR1-5-OP-RU$^+$ TCRβ$^+$ cells

| | TRAV | CDR3α | TRAJ | TRBV | CDR3β | TRBJ | Freq. |
|---|---|---|---|---|---|---|---|
| WT Expanded Splenic MR1-5-OP-RU$^+$ TCRβ$^+$ cells | 1 | CAVKDSNYQLIW | 33 | 2 | CASSQDLGTGVERLFF | 1-4 | 1 |
| | 1 | CAVRDSNYQLIW | 33 | 2 | CASSQGDWGGSYEQYF | 2-7 | 1 |
| | 1 | CAVRNSNYQLIW | 33 | 4 | CASSPDWGSAETLYF | 2-3 | 1 |
| | 1 | CAVRDSNYQLIW | 33 | 13-2 | CASGAPQGGAETLYF | 2-3 | 1 |
| | 1 | CAVRDTXYXLXW | 33 | 13-2 | CASGERTGLYEQYF | 2-7 | 1 |
| | 1 | CAVRDTNYQLIW | 33 | 13-3 | CASSXETGKDXEQXF | 2-1 | 1 |
| | 1 | CAVRDSNYQLIW | 33 | 13-3 | CASSGEGGGYAEQFF | 2-1 | 1 |
| | 1 | CAVMDSNYQLIW | 33 | 13-3 | CASSAPGQGNTGQLYF | 2-2 | 1 |
| | 1 | CAIMDSNYQLIW | 33 | 13-3 | CASGLGGDAETLYF | 2-3 | 1 |
| | 1 | CAVKDSNYQLIW | 33 | 13-3 | CASSGTGGSAETLYF | 2-3 | 1 |
| | 1 | CAVMDSNYQLIW | 33 | 13-3 | CASSAPGQGNTGQLYF | 2-4 | 1 |
| | 1 | CAVRDSNYQLXW | 33 | 29 | CASSLEDRVYAEXXF | 2-1 | 1 |
| | 1 | CAVKDSNYQLXW | 33 | | n.d. | | 1 |
| | 1 | CXGRDSXXQLIW | 33 | | n.d. | | 1 |
| | 1 | CAEMDSNYQLIW | 33 | | n.d. | | 1 |
| | 1 | CAVRDSNYQLIW | 33 | 13-2 | 2 sequences | | 1 |
| Traj33 KO Expanded Splenic MR1-5-OP-RU$^+$ TCRβ$^+$ cells | 1 | CAVRDGGYKLTF | 9 | 13-3 | CASSGTENTEVFF | 1-1 | 1 |
| | 1 | CAVRDGGYKLTF | 9 | 13-2 | CASGTGESAETLYF | 2-3 | 6 |
| | 1 | CAVRDGGYKLTF | 9 | 13-2 | CASGDAPGLGAETLYF | 2-3 | 1 |
| | 1 | CAVRDGGYKLTF | 9 | | n.d. | | 1 |
| | 1 | CAVMTGGYKVVF | 12 | 13-2 | CASGDAKDWGDAEQFF | 2-1 | 3 |
| | 1 | CAVRDGGYKVVF | 12 | 13-2 | CASGEGGDNYAEQFF | 2-1 | 2 |
| | 1 | CAVRDGGYKVVF | 12 | 13-2 | CASGDDGGAETLYF | 2-3 | 4 |
| | 1 | CAVRDGGYKVVF | 12 | 13-2 | CASGDDRGGAETLYF | 2-3 | 3 |
| | 1 | CAVRDGGYKVVF | 12 | 13-3 | CASSETDNNQAPXF | 1-5 | 2 |
| | 1 | CAVRDGGYKVVF | 12 | 13-2 | 2 sequences | 2-3 | 1 |
| | 1 | CAVRDGGYKVVF | 12 | 13-3 | 2 sequences | 2-3 | 1 |
| | 1 | CAVRDGGYKVVF | 12 | 13-3 | 2 sequences | | 1 |
| | 1 | CAVRDGGYKVVF | 12 | 13-3 | 2 sequences | | 1 |
| | 1 | CAVRDGGYKVVF | 12 | | n.d. | | 1 |
| | 1 | CAVQTGNYKYVF | 40 | 1 | CTCSADPDRDGNTLYF | 1-3 | 1 |
| | 1 | CAVRDGNYKYXF | 40 | 13-3 | CASSEQGGGAETLYF | 2-3 | 1 |
| | 1 | n.d. | 12 | 13-2 | CASGDAKDWGDAEQFF | 2-1 | 2 |
| | 1 | n.d. | 12 | | n.d. | | 1 |
| | 1 | n.d. | 40 | 13-3 | CASSNYNSPLYF | 1-6 | 1 |
| | 1 | 2 sequences | 40 | 13-3 | CASSEQGGGAETLYF | 2-3 | 6 |

n.d. = not determined due to poor sequencing trace data.
Where multiple sequences were identified, both are described as (i) and (ii).
Where 2 overlapping sequenced were present, ‘2 sequences’ is indicated.
Where nucleotide sequence was unclear, amino acid is designated as ‘X’.

**Supplementary Table 2.** TCR sequences of MR1-5-OP-RU$^{+}$ TCRβ$^{+}$ cells from enriched WT thymus

| TRAV | CDR3α | TRAJ | TRBV | CDR3β | TRBJ | Freq. |
|---|---|---|---|---|---|---|
| 1 | CAVRDREDMGYXLTF | 9 | 13-3 | CASSDSYSYNSPLYF | 1-6 | 1 |
| 1 | CAVRDGGYKLTF | 9 | 13-3 | CASSDPGGHEQYF | 2-7 | 1 |
| 1 | CAXXDSNYXLIW | 33 | 1 | CTCSADPHSGNTLYF | 1-3 | 1 |
| 1 | CAVRDSNYQLIW | 33 | 1 | CTCSAEEEGGRDTQYF | 2-5 | 1 |
| 1 | CAVRDSNYQLIW | 33 | 2 | CASSLEYNYAEQFF | 2-1 | 1 |
| 1 | CAVKDSNYQLIW | 33 | 4 | CASSSDWGGAETLYF | 2-3 | 2 |
| 1 | CXVRDSXYQLXW | 33 | 4 | CASSPGWGGAGEQYF | 2-7 | 1 |
| 1 | CAVRDSNYQLIW | 33 | 5 | CASSQGLGGAETLYF | 2-3 | 1 |
| 1 | CAVRDSNYQLIW | 33 | 5 | CASSQESGGPETLYF | 2-3 | 1 |
| 1 | CAVRDSNYQLIW | 33 | 5 | CASSQEVGAYYEQYF | 2-7 | 1 |
| 1 | CAVMDSNYQLIW | 33 | 12-2 | CASSPGTGDRETLYF | 2-3 | 1 |
| 1 | CAVRDSNYQLIW | 33 | 13-1 | CASSDAREYAEQFF | 2-1 | 1 |
| 1 | CAVKDSNYQLIW | 33 | 13-1 | CASSEGWDGAETLYF | 2-3 | 1 |
| 1 | CAVRDSNYQLIW | 33 | 13-1 | CASSDAHYEQYF | 2-7 | 1 |
| 1 | CAVRDSNYQLIW | 33 | 13-1 | CASGGDIYEQYF | 2-7 | 1 |
| 1 | CAVRDSNYQLIW | 33 | 13-1 | CASSERGQYEQYF | 2-7 | 1 |
| 1 | CAVRDSNYQLIW | 33 | 13-1 | CASSEQDWGGYEQYF | 2-7 | 1 |
| 1 | CAVRDSNYQLIW | 33 | 13-2 | CASGTGDNQAPLF | 1-5 | 1 |
| 1 | CAVMDSNYQLIW | 33 | 13-3 | CASSPGGAPAEQFF | 2-1 | 1 |
| 1 | CAVRDSNYQLIW | 33 | 13-3 | CASSDARGQDTQYF | 2-5 | 1 |
| 1 | CAVRDSNYQLIW | 33 | 13-2 | CASGEHNQAPLF | 1-5 | 1 |
| 1 | CAVRDSNYQLIW | 33 | 13-2 | CASGDGLGDNYAEQFF | 2-1 | 1 |
| 1 | CAVKDSNYQLIW | 33 | 13-2 | CASGDAGGHTGQLYF | 2-2 | 1 |
| 1 | CAVRDSNYQLIW | 33 | 13-2 | CASGDAGAAETLYF | 2-3 | 1 |
| 1 | CAVRDSNYQLIW | 33 | 13-2 | CASGDATGGGAETLYF | 2-3 | 1 |
| 1 | CAVRDSNYQLIW | 33 | 13-2 | CASGEIGGAETLYF | 2-3 | 1 |
| 1 | CAVRDSNYQLIW | 33 | 13-2 | CASGEGGRGAETLYF | 2-3 | 1 |
| 1 | CAVRDSNYQLIW | 33 | 13-2 | CASGGTGGADEQYF | 2-7 | 1 |
| 1 | CAVRDSNYQLIW | 33 | 13-2 | CASGRDNYEQYF | 2-7 | 1 |
| 1 | CAVRDSNYQLIW | 33 | 13-3 | CASSDGTGAPEVFF | 1-1 | 1 |
| 1 | CAVRDSNYQLIW | 33 | 13-3 | CASGKGQEGSDYTF | 1-2 | 1 |
| 1 | CAVRDSNYQLIW | 33 | 13-3 | CASSDGENSPLYF | 1-6 | 1 |
| 1 | CAVRDSNYQLIW | 33 | 13-3 | CASSDELGYAEQFF | 2-1 | 1 |
| 1 | CAVRDSNYQLIW | 33 | 13-3 | CASTPGQNYAEQFF | 2-1 | 1 |
| 1 | CAVRDSNYQLIW | 33 | 13-3 | CASSGDRDYAEQFF | 2-1 | 1 |
| 1 | CAVRDSNYQLIW | 33 | 13-3 | CASSHRGHTGQLYF | 2-2 | 1 |
| 1 | CAVRDSNYQLIW | 33 | 13-3 | CASSDWGGANTGQLYF | 2-2 | 1 |
| 1 | CAVRDSNYQLIW | 33 | 13-3 | CASSPTGIGQLYF | 2-2 | 1 |
| 1 | CAVRDRDYQLIW | 33 | 13-3 | CASSYRVSTGQLYF | 2-2 | 1 |
| 1 | CAVRDSNYQLIW | 33 | 13-3 | CASSEPGQGAETLYF | 2-3 | 1 |
| 1 | CAVRDSNYQLIW | 33 | 13-3 | CASSSDSSSAETLYF | 2-3 | 1 |
| 1 | CAVRDSNYQLIW | 33 | 13-3 | CASSRDSKDTQYF | 2-5 | 1 |
| 1 | CAVRDSNYQLIW | 33 | 13-3 | CASSDARNQDTQYF | 2-5 | 1 |
| 1 | CAVRDSNYQLIW | 33 | 19 | CASSIATENTEVFF | 1-1 | 1 |
| 1 | CAVRDSNYQLIW | 33 | 19 | CASSMEGGNSPLYF | 1-6 | 1 |
| 1 | CAVRDSNYQLIW | 33 | 19 | CASSIGADTGQLYF | 2-2 | 1 |
| 1 | CAVRDSNYQLIW | 33 | 19 | CASSISGGADAETLYF | 2-3 | 1 |
| 1 | CAVRDSNYQLIW | 33 | 19 | CASSMGQGSAETLYF | 2-3 | 1 |
| 1 | CAVRDSNYQLIW | 33 | 19 | CASSMTGGNAETLYF | 2-3 | 1 |
| 1 | CAVRDSNYQLIW | 33 | 19 | CASSPHREGAETLYF | 2-3 | 1 |
| 1 | CAVMDSNYQLIW | 33 | 19 | CASSMLGGSSQNTLYF | 2-4 | 1 |
| 1 | CAVRDSNYQLIW | 33 | 19 | CASEDWGGYEQYF | 2-7 | 1 |
| 1 | CAVRDSNYQLIW | 33 | 13-3 | n.d. | | 1 |

n.d. = not determined due to poor sequencing trace data.
Where nucleotide sequence was unclear, amino acid is designated as 'X'.

**Supplementary Table 3.** Additional TCRα sequences of MR1-5-OP-RU+ TCRβ+ cells from enriched WT thymus

| TRAV | CDR3α | TRAJ | Freq. |
|---|---|---|---|
| 1 | CAVRDSNYXLIW | 33 | 1 |
| 1 | CAVRDSNYQLIW | 33 | 1 |
| 1 | CAVRDSNYQLIW | 33 | 1 |
| 1 | CAVRDSNYQLIW | 33 | 1 |
| 1 | CAVTDSNYQLIW | 33 | 1 |
| 1 | CAVRDSNYQLIW | 33 | 1 |
| 1 | CAVRDSNYQLIW | 33 | 1 |
| 1 | CAVRDSNYXLIW | 33 | 1 |
| 1 | CAVRDSNYQLIW | 33 | 1 |
| 1 | CAVADSNYQLIW | 33 | 1 |
| 1 | CAVRDSNYQLIW | 33 | 1 |
| 1 | CAVRDXNYXLIW | 33 | 1 |
| 1 | CAVMDSNYQLIW | 33 | 1 |
| 1 | CAVRDSNYXLIW | 33 | 1 |
| 1 | CAVRDSNYXLIW | 33 | 1 |
| 1 | CAVMDSNYQLIW | 33 | 1 |
| 1 | CAVRDSNYQLIW | 33 | 1 |
| 1 | CAVRDSNYQLIW | 33 | 1 |
| 1 | CAVRDSNYQLIW | 33 | 1 |
| 1 | CAVRXSXYXLIW | 33 | 1 |
| 1 | CAVRDSNYQLIW | 33 | 1 |
| 1 | CAVMDSNYXLIW | 33 | 1 |
| 1 | CAVRDSNYQLIW | 33 | 1 |
| 1 | CAVRDSNYQLIW | 33 | 1 |
| 1 | CAVRDSNYQLIW | 33 | 1 |
| 1 | CAGMDSNYQLIW | 33 | 1 |
| 1 | CAVRDSNYQLIW | 33 | 1 |
| 1 | CAVLDSNYXLIW | 33 | 1 |
| 1 | CAVRDSNYQLIW | 33 | 1 |
| 1 | CAVRDSNYQLIW | 33 | 1 |
| 1 | CAVRDSNYXLIW | 33 | 1 |
| 1 | CAVRDSNYQLIW | 33 | 1 |
| 1 | CAVRDSNYQLIW | 33 | 1 |
| 1 | CAVRDSNYQLIW | 33 | 1 |
| 1 | CAVLDSNYQLIW | 33 | 1 |
| 1 | CAVRDSNYQLIW | 33 | 1 |
| 1 | CAVRXSNYQLIW | 33 | 1 |
| 1 | CAVRXSNYQLIW | 33 | 1 |
| 1 | CAVRDSNYQLIW | 33 | 1 |
| 1 | CAVRDSNYQLIW | 33 | 1 |
| 1 | CAVRDSNYQLIW | 33 | 1 |
| 1 | CAVRDSNYQLIW | 33 | 1 |
| 1 | CAVRDSNYQLIW | 33 | 1 |
| 1 | CAVRDSNYQLIW | 33 | 1 |
| 1 | CAVRDSNYQLIW | 33 | 1 |
| 1 | CAVRDSNYXLIW | 33 | 1 |
| 1 | CAVRDSNYQLIW | 33 | 1 |
| 1 | CAVRDSNYQLIW | 33 | 1 |
| 1 | CAVRDSNYQLIW | 33 | 1 |
| 1 | CAVRDSNYQLIW | 33 | 1 |
| 1 | CAVRDSNYQLIW | 33 | 1 |
| 1 | CAVRXSNYQLIW | 33 | 1 |
| 1 | CAVLDSNYQLIW | 33 | 1 |
| 1 | CAVVDSNYQLIW | 33 | 1 |
| 1 | CAVINSNYQLIW | 33 | 1 |
| 1 | CAVRDSNYQLIW | 33 | 1 |
| 1 | CAVRDSNYQLIW | 33 | 1 |
| 1 | CAVRDSNYQLIW | 33 | 1 |

Where nucleotide sequence was unclear, amino acid is designated as ‘X’.

**Supplementary Table 4.** TCR sequences of MR1-5-OP-RU$^{+}$ TCRβ$^{+}$ cells from enriched *Traj33*$^{-/-}$ thymus

| | TRAV | CDR3α | TRAJ | TRBV | CDR3β | TRBJ | Freq. |
|---|---|---|---|---|---|---|---|
| TRAV1$^{+}$ | 1 | CAVSGGNYKPTF | 6 | 13-3 | CASSDSLYAEQFF | 2-1 | 1 |
| | 1 | CAVRDGGYKLTF | 9 | 13-2 | CASGDAGGGAETLYF | 2-3 | 1 |
| | 1 | CAVRDGGYKLTF | 9 | 13-2 | CASGDAGVGAETLYF | 2-3 | 1 |
| | 1 | CAVRDEGYKLTF | 9 | 13-2 | CASGDNWGGVGAETLYF | 2-3 | 1 |
| | 1 | CAVRDGGYKLTF | 9 | 13-3 | CASSEGTAYAEQFF | 2-1 | 1 |
| | 1 | CAVRDGGYKLTF | 9 | 13-3 | CASSPGGGAGQLYF | 2-2 | 1 |
| | 1 | CAVRDGGYKLTF | 9 | 13-3 | CASSGGGDAETLYF | 2-3 | 1 |
| | 1 | CAVRDGGYKLTF | 9 | 13-3 | CASSDDGGGAETLYF | 2-3 | 2 |
| | 1 | CAVRDGGYKLTF | 9 | 13-3 | CASSDAGEYEQYF | 2-7 | 2 |
| | 1 | CAVSDMGYKLTF | 9 | 19 | CASSIGGGRGAETLYF | 2-3 | 1 |
| | 1 | CAARTGGYKVVF | 12 | 13-2 | CASGDNWDGYSDYTF | 1-2 | 1 |
| | 1 | CAVRDGGYKVVF | 12 | 13-3 | CASSETGDTNSDYTF | 1-2 | 1 |
| | 1 | CAVSTNAYKVIF | 30 | 2 | CASSPNWGPYAEQFF | 2-1 | 1 |
| | 1 | CAVRDGAYKVIF | 30 | 13-3 | CASSPGQENSDYTF | 1-2 | 1 |
| | 1 | CAVRDDAYKVIF | 30 | 13-3 | CASSGTEYAEQFF | 2-1 | 1 |
| | 1 | CAVLTGNYKYVF | 40 | 13-3 | CASSLTDQDTQYF | 2-5 | 1 |
| | 1 | CAVMTGNYKYVF | 40 | 13-3 | CASSDAREVRDTQYF | 2-5 | 1 |
| | 1 | CAVNTGNYKYVF | 40 | 13-2 | CASGDXWGGVGAXTLYF | 2-3 | 1 |
| TRAV1$^{-}$ | 3-4 | CAVDWTGNYKYVF | 40 | 12-1 | CASSLGQVPNERLFF | 1-4 | 1 |
| | 3D/N | CAVKAGGYKVVF | 12 | 13-1 | CASSDGWGDTQYF | 2-5 | 1 |
| | 3D/N | CAVGSNSGTYQRF | 13 | 13-2 | CASGDAQFWGQDTQYF | 2-5 | 1 |
| | 3D/N | CAVSEGQGGRALIF | 15 | 29 | CASSLNRGNNQXPLF | 1-5 | 1 |
| | 6/X | CAXSDRGGGSXKLXF | 57 | 5 | CASSHWGSANTGQLYF | 2-2 | 1 |
| | 6D/N | CAPHNTNTGKLTF | 27 | 31 | CAWSSWGGGSAETLYF | 2-3 | 1 |
| | 6D/N | CALSESNNRIFF | 31 | 13-1 | CASSKGEGANQAPLF | 1-5 | 1 |
| | 9N-3 | CAVPLGGNTGKLIF | 37 | 14 | CASSFGDYAEQFF | 2-1 | 1 |
| | 15D-2 | CALSELPGGYKVVF | 12 | 13-3 | CASSGTGASYNSPLYF | 1-6 | 1 |
| | 16D/N | CAMREGSGSFNKLTF | 4 | 2 | CASSQDLGASAETLYF | 2-3 | 1 |
| | 16D/N | CAMREANSAGNKLTF | 17 | 5 | CASSQDRLGVGAETLYF | 2-3 | 1 |
| | 16D | CAMRDRGSALGRLHF | 18 | 1 | CTCSAGNTEVFF | 1-1 | 1 |
| | 16D | CAMREGEGRGSALGRLHF | 18 | 1 | CTCSADPRWDTEVFF | 1-1 | 1 |
| | 16D/N | CAMRDRGSALGRLHF | 18 | 13-2 | CASGDAGTFYAEQFF | 2-1 | 1 |
| | 16D | CAMRDRGSALGRLHF | 18 | 13-2 | CASGDWAFSYEQYF | 2-7 | 1 |
| | 16D/N | CAMRDRGSALGRLHF | 18 | 13-2 | CASGDWGYQDTQYF | 2-5 | 2 |
| | 16D | CAMRDRGSALGRLHF | 18 | 13-3 | CASSPGGGAGQLYF | 2-2 | 1 |
| | 16D/N | CAMREPASSGSWQLIF | 22 | 2 | CASSQEWTENTGQLYF | 2-2 | 1 |
| | 16D/N | CAMRESSSGSWQLIF | 22 | 12-2 | CASSLDREYSAETLYF | 2-3 | 1 |
| | 16D/N | CAMREYAQGLTF | 26 | 31 | CAWSLVGRRRLFF | 1-4 | 1 |
| | 16D/N | CAMANYAQGLTF | 26 | 31 | CAWKGWGGAGAETLYF | 2-3 | 1 |
| | 16D/N | CAMREPSSNTNKVVF | 34 | 16 | CASSLGGADTQYF | 2-5 | 1 |
| | 16D/N | CAMRDTGNYKYVF | 40 | 4 | CASSPDWGGYAEQFF | 2-1 | 1 |
| | 16D/N | CAMRESSSFSKLVF | 50 | 31 | CAXSWGVAETLYF | 2-3 | 1 |
| | 16D | CAMRDTGANTGKLTF | 52 | 13-2 | CASGGRGSPLYF | 1-6 | 1 |

Where nucleotide sequence was unclear, amino acid is designated as 'X'.
TRAV3D/N = could not determine if TRAV3D or 3N
TRAV6-X = could not determine which TRAV6 gene family member
TRAV6D/N = could not determine if TRAV6D or 6N
TRAV16D/N = could not determine if TRAV16D or 16N

**Supplementary Table 5.** TCRα sequences of *in vivo* expanded pulmonary MR1-5-OP-RU$^{+}$ TCRβ$^{+}$ cells post *L. longbeachae* infection.

| Lung | TRAV | CDR3α | TRAJ | Freq. |
|---|---|---|---|---|
| WT | 1 | CAVRDSNYQLIW | 33 | 15 |
| | 1 | CAVKDSNYQLIW | 33 | 2 |
| | 1 | CAVLDSNYQLIW | 33 | 1 |
| | 1 | CAVMDSNYQLIW | 33 | 1 |
| | 1 | CAVRDXNYXLIW | 33 | 1 |
| | 3D/N | CAVRETGFASALTF | 35 | 1 |
| Traj33 KO | 1 | CAVRDEGYKLTF | 9 | 13 |
| | 1 | CAVRDGGYKLTF | 9 | 6 |
| | 1 | CAAWTGGYKVVF | 12 | 3 |
| | 1 | CAVCDGGYKVVF | 12 | 2 |
| | 1 | CAVIDGGYKVVF | 12 | 2 |
| | 1 | CAVRDGGYKLTF | 12 | 2 |
| | 1 | CAVATNAYKVIF | 30 | 8 |
| | 1 | CAVRDGAYKVIF | 30 | 3 |
| | 1 | CXVRXXXXXXXF | 30 | 1 |
| | 7-4 | CAASVATASLGKLQF | 24 | 1 |

Where nucleotide sequence was unclear, amino acid is designated as ‘X’.
TRAV3D/N = could not determine if TRAV3D or 3N

**Supplementary Table 6.** List of antibodies used for flow cytometry.

| **Antigen** | **Reactivity** | **Clonev fv** | **Fluorophore** | **Dilution** | **Vendor** | **Catalogue #** |
|---|---|---|---|---|---|---|
| CD3 | Human | UCHT1 | BUV395 | 1:50 | BD Pharmingen | 563546 |
| CD3 | Human | UCHT1 | PE | 1:50 | BD Pharmingen | 555333 |
| CD3 | Human | UCHT1 | AF700 | 1:50 | BD Pharmingen | 557943 |
| CD4 | Human | SK3 | BUV496 | 1:50 | BD Pharmingen | 564651 |
| CD8α | Human | SK1 | BUV805 | 1:400 | BD Pharmingen | 564912 |
| CD14 | Human | MϕP9 | APC-Cy7 | 1:50 | BD Pharmingen | 557831 |
| CD19 | Human | SJ25C1 | APC-Cy7 | 1:50 | BD Pharmingen | 557791 |
| CD26 | Human | BA-5b | PE-Cy7 | 1:100 | Biolegend | 302713 |
| CD45 | Human | 2D1 | AF700 | 1:100 | Biolegend | 368514 |
| CD161 | Human | HP-3G8 | BV650 | 1:50 | Biolegend | 339916 |
| CD218α | Human | H44 | APC | 1:50 | Biolegend | 17-7183-42 |
| CD218α | Human | H44 | PE | 1:50 | eBioscience | 12-7183-42 |
| TCRγδ | Human | 11F2 | FITC | 1:50 | BD Pharmingen | 347903 |
| TRAV1-2 | Human | 3C10 | BV711 | 1:50 | Biolegend | 351732 |
| TRAV1-2 | Human | 3C10 | APC | 1:50 | Biolegend | 351708 |
| PLZF | Human and Mouse | Mags.21F7 | PE | 1:50 (Hu) 1:200 (Mo) | eBioscience | 12-9320-80 |
| CD3 | Mouse | 145-2C11 | BV786 | 1:100 | BD Pharmingen | 564379 |
| CD4 | Mouse | RM4-5 | BUV395 | 1:200 | BD Pharmingen | 740208 |
| CD8 | Mouse | 53.6-7 | BUV805 | 1:300 | BD Pharmingen | 564920 |
| CD44 | Mouse | 1M7 | AF700 | 1:200 | eBioscience | 56-0441-82 |
| B220 | Mouse | RA3-6B2 | BUV496 | 1:200 | BD Pharmingen | 564662 |
| TCRβ | Mouse | H57-597 | APC-Cy7 | 1:200 | BD Pharmingen | 560656 |
| TCRγδ | Mouse | GL3 | FITC | 1:100 | eBioscience | 11-5711-82 |
