# Supplementary material for: Diverse MR1-restricted T cells in mice and humans
Source: Nat Commun. 2019 May 21;10:2243. doi: 10.1038/s41467-019-10198-w (PMC6529461; doi:10.1038/s41467-019-10198-w)
Supplement: Supplementary file 3 — Reporting Summary [file 41467_2019_10198_MOESM3_ESM.pdf]

nature research

Corresponding author(s): Dale Godfrey
Last updated by author(s): Apr 15, 2019

Reporting Summary

Nature Research wishes to improve the reproducibility of the work that we publish. This form provides structure for consistency and transparency in reporting. For further information on Nature Research policies, see Authors & Referees and the Editorial Policy Checklist.

Statistics

For all statistical analyses, confirm that the following items are present in the figure legend, table legend, main text, or Methods section.

n/a	Confirmed	
☐	☒	The exact sample size (n) for each experimental group/condition, given as a discrete number and unit of measurement
☐	☒	A statement on whether measurements were taken from distinct samples or whether the same sample was measured repeatedly
☐	☒	The statistical test(s) used AND whether they are one- or two-sided<br>*Only common tests should be described solely by name; describe more complex techniques in the Methods section.*
☐	☒	A description of all covariates tested
☐	☒	A description of any assumptions or corrections, such as tests of normality and adjustment for multiple comparisons
☐	☒	A full description of the statistical parameters including central tendency (e.g. means) or other basic estimates (e.g. regression coefficient) AND variation (e.g. standard deviation) or associated estimates of uncertainty (e.g. confidence intervals)
☐	☒	For null hypothesis testing, the test statistic (e.g. F, t, r) with confidence intervals, effect sizes, degrees of freedom and P value noted<br>*Give P values as exact values whenever suitable.*
☒	☐	For Bayesian analysis, information on the choice of priors and Markov chain Monte Carlo settings
☒	☐	For hierarchical and complex designs, identification of the appropriate level for tests and full reporting of outcomes
☒	☐	Estimates of effect sizes (e.g. Cohen's d, Pearson's r), indicating how they were calculated

Our web collection on statistics for biologists contains articles on many of the points above.

Software and code

Policy information about availability of computer code

Data collection	N/A
Data analysis	N/A

For manuscripts utilizing custom algorithms or software that are central to the research but not yet described in published literature, software must be made available to editors/reviewers. We strongly encourage code deposition in a community repository (e.g. GitHub). See the Nature Research guidelines for submitting code & software for further information.

Data

Policy information about availability of data

All manuscripts must include a data availability statement. This statement should provide the following information, where applicable:

- Accession codes, unique identifiers, or web links for publicly available datasets
- A list of figures that have associated raw data
- A description of any restrictions on data availability

N/A

Field-specific reporting

Please select the one below that is the best fit for your research. If you are not sure, read the appropriate sections before making your selection.

☒ Life sciences ☐ Behavioural & social sciences ☐ Ecological, evolutionary & environmental sciences

For a reference copy of the document with all sections, see nature.com/documents/nr-reporting-summary-flat.pdf

Life sciences study design

All studies must disclose on these points even when the disclosure is negative.

Sample size	For mouse experiments (Figure 1,2,3 & 5), a total of at least 4-8 mice per strain were used and carried over at least two independent experiments. For human blood experiments, at least 15 donor samples were analysed across 3 independent experiments.
Data exclusions	No data was excluded from the analyses, including TRAV3 cell line data discussed in review that is now incorporated into Supplementary Figure 5.
Replication	All attempts at replication were successful.
Randomization	N/A
Blinding	Blinding was not relevant to our study.

Reporting for specific materials, systems and methods

We require information from authors about some types of materials, experimental systems and methods used in many studies. Here, indicate whether each material, system or method listed is relevant to your study. If you are not sure if a list item applies to your research, read the appropriate section before selecting a response.

Materials & experimental systems

n/a	Involved in the study	
☐	☒	Antibodies
☐	☒	Eukaryotic cell lines
☒	☐	Palaeontology
☐	☒	Animals and other organisms
☐	☒	Human research participants
☒	☐	Clinical data

Methods

n/a	Involved in the study	
☒	☐	ChIP-seq
☐	☒	Flow cytometry
☒	☐	MRI-based neuroimaging

Antibodies

Antibodies used	As per supplementary Table 4.
Validation	All antibodies used in this manuscript are commercially available, validated by manufacturers and widely accepted in the field for their intended use in this study. Antibodies were validated to be working by staining of relevant positive control samples prior to embarking upon formal experiments published here.

Eukaryotic cell lines

Policy information about cell lines

Cell line source(s)	ATCCC
Authentication	None of the cell lines used were authenticated
Mycoplasma contamination	HEK293t cell line tested negative for mycoplasma contamination.
Commonly misidentified lines (See ICLAC register)	N/A

Animals and other organisms

Policy information about studies involving animals; ARRIVE guidelines recommended for reporting animal research

Laboratory animals	Stated in page 18 in manuscript.
Wild animals	The study did not involve wild animals.
Field-collected samples	The study did not involve field-collected samples.
Ethics oversight	Stated in page 18 in manuscript.

Note that full information on the approval of the study protocol must also be provided in the manuscript.

Human research participants

Policy information about studies involving human research participants

Population characteristics	Human blood was sourced from individuals in Melbourne, Australia who were were deemed eligeable to donoate by the Australian Red Cross. This included such thing as, butnot limited to, being aged between 18 and 70 years of age, had no recent tattoos or surgeries, and were deemed healthy at the time of donation. Samples tested negative for HIV, Hepatitis B and Hepatitis C.
Recruitment	Blood samples were recruited at random from Australian Red Cross blood bank donors.
Ethics oversight	Stated in page 18 in manuscript.

Note that full information on the approval of the study protocol must also be provided in the manuscript.

Flow Cytometry

Plots

Confirm that:

☐ The axis labels state the marker and fluorochrome used (e.g. CD4-FITC).

☒ The axis scales are clearly visible. Include numbers along axes only for bottom left plot of group (a 'group' is an analysis of identical markers).

☒ All plots are contour plots with outliers or pseudocolor plots.

☒ A numerical value for number of cells or percentage (with statistics) is provided.

Methodology

Sample preparation	Stated in pages 18-19 in manuscript.
Instrument	Stated in page 21 in manuscript.
Software	Stated in pages 21-22 in manuscript.
Cell population abundance	Post-sort fractions for Figure 3A are included below pre-sort plots.
Gating strategy	As per supplementary Figure 7.

☒ Tick this box to confirm that a figure exemplifying the gating strategy is provided in the Supplementary Information.
